# Impact of large‐loop excision of the transformation zone on cervical morphology: A prospective observational study

**DOI:** 10.1002/ijgo.70447

**Published:** 2025-08-25

**Authors:** Flávia Zero Soares, Helmer Herren, Patrícia Pereira dos Santos Melli, Marília Veccechi Bijos Zaccaro, Silvana Maria Quintana

**Affiliations:** ^1^ Department of Gynecology and Obstetrics, Ribeirão Preto Medical School University of Sao Paulo Ribeirão Preto Brazil

**Keywords:** cervical dimensions, cervical regeneration, large‐loop excision of the transformation zone, ultrasonographic assessment

## Abstract

**Objectives:**

To evaluate the impact of large‐loop excision of the transformation zone (LLETZ) on cervical dimensions, to identify influencing factors on cervical regeneration and to verify the concordance between ultrasonographic techniques for assessing cervical volume.

**Methods:**

This was a prospective observational study conducted at the University of São Paulo Clinical Hospital from May 2022 to April 2023. Ultrasonographic measurements of cervical length and volume were obtained before LLETZ and at 3 and 6 months post‐procedure. The influence of variables on the regenerative process was evaluated using generalized linear regression models, and the concordance between volume measurements using the cylinder formula and VOCAL (Virtual Organ Computer‐Aided Analysis) software was assessed.

**Results:**

The mean cervical length decreased from 32.3 mm to 30.3 mm at 3 months but increased to 31.7 mm at 6 months. The volume calculated by the cylinder formula decreased from 15.6 cm^3^ to 11.6 cm^3^ at 3 months, rising to 12.3 cm^3^ at 6 months. Similarly, the volume measured by VOCAL dropped from 16.0 cm^3^ to 11.8 cm^3^ at 3 months and increased to 13.1 cm^3^ at 6 months. The volume of excised tissue influences cervical length, whereas parity affects volume regeneration. Ultrasonography methods for assessing cervical volume showed high concordance.

**Conclusion:**

There is a regenerative process of the cervix after LLETZ, complete in terms of length and partial in volume. The volume of the excised tissue and parity influence the regenerative process. Ultrasonographic assessments of cervical volume by two‐ or three‐dimensional methods are comparable.

## 1 INTRODUCTION

Large‐loop excision of the transformation zone (LLETZ) has been a widely used method for the treatment of precancerous cervical lesions for decades. This procedure is highly effective and offers significant advantages, including the ability to provide histologic confirmation and margin assessment.^1^ However, growing evidence has highlighted the risk of adverse obstetric outcomes in patients undergoing LLETZ.^2^, ^3^, ^4^, ^5^, ^6^ Although cervical cancer is largely preventable, it remains a significant global public health concern, ranking as the fourth leading cause of cancer‐related deaths among women worldwide.^7^ Efforts to eliminate cervical cancer focus on improving prevention, screening, and treatment strategies for precancerous lesions.^8^ Advances in screening methods are expected to increase detection and treatment rates.^9^

Precancerous lesions primarily affect young women of reproductive age, many of whom have not yet had children.

The highest prevalence is found among women from low‐ and middle‐income countries, who face considerable challenges from diagnosis to treatment, and are particularly vulnerable to the potential reproductive consequences of the treatment of these lesions.^10^ Without a comprehensive understanding of these impacts, additional complications may arise, including in reproductive health, psychosocial, economic, and quality of life issues. Therefore, it is crucial to develop a thorough understanding of the impact of LLETZ on cervical morphology, the dynamics of the regenerative process, the contributing factors, and the optimal methods for assessment.

## 2 MATERIALS AND METHODS

This prospective, longitudinal, observational study was conducted at the University of São Paulo Clinical Hospital between May 2022 and April 2023 to assess cervical regeneration following LLETZ. Patients were consecutively recruited from the Gynecology Department, and all provided written informed consent before participation.

Ultrasonographic measurements of cervical length and volume were obtained at three time points: before LLETZ, 3 months post‐procedure, and 6 months post‐procedure. The primary outcome measures were changes in cervical length and volume over time. Secondary analyses included the influence of excised tissue dimensions, parity, and type of loop electrode used on cervical regeneration, as well as the concordance between volume measurements obtained using the cylinder formula and VOCAL (Virtual Organ Computer‐Aided Analysis) software.

The study was approved by the Institutional Review Board (Approval No. 5.439.786).

Inclusion criteria were patients undergoing LLETZ at the University of São Paulo Clinical Hospital with no previous cervical surgeries and with availability of cervical ultrasound measurements at baseline and at least one follow‐up time point (3 or 6 months).

Exclusion criteria were as follows (1) history of cervical malignancy; (2) pregnancy during the study period; (3) inadequate ultrasound images; and (4) absence of follow‐up measurements at both 3 and 6 months.

LLETZ was performed under colposcopic visualization and local anesthesia, using semilunar or triangular tungsten loop electrodes connected to a high‐frequency electrosurgical unit operating at 4000 MHz and 220 V. The loop size and type were chosen based on lesion size and transformation zone type. Power settings ranged from 60 to 80 W in blended cutting mode. Hemostasis was achieved selectively at identified bleeding points. Procedures were conducted by gynecologists in fellowship training in lower genital tract pathology or by senior residents, all under the supervision of experienced attending physicians. The volume of the excised specimen was assessed according to the Archimedes principle,^11^ and its length was measured and documented in the histopathology laboratory after preservation in formalin.

Ultrasound measurements were performed with the patient in the gynecologic position after bladder emptying. The equipment used was a Samsung WS80A ultrasound system equipped with a 3‐ to 10‐MHz transvaginal probe.

**FIGURE 1:**
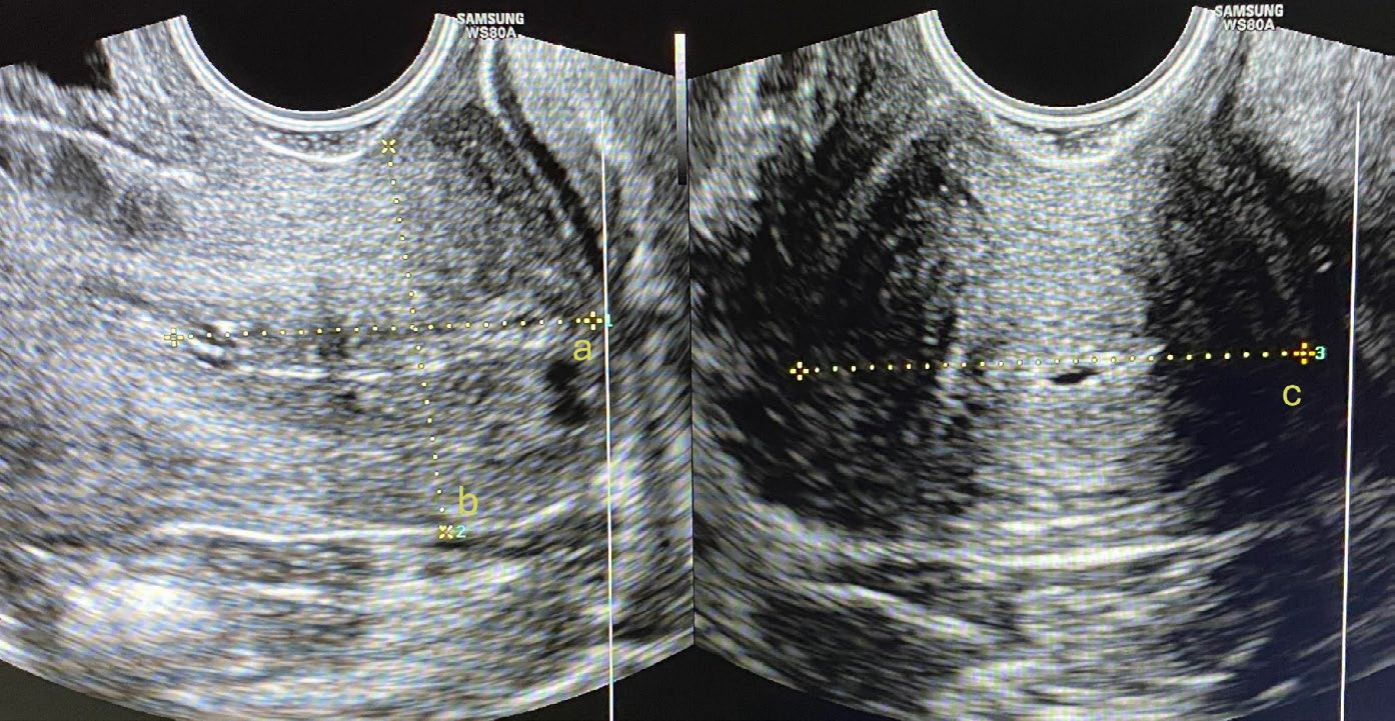
Cervical measurements using 2D ultrasound. (a) Cervical length and (b) anteroposterior (AP) diameter measured in the sagittal plane, and (c) transverse (T) diameter measured in the transverse plane.

Cervical length, anteroposterior diameter, and transverse diameter were measured in two‐dimensional (2D) mode. The cervical length was determined in the midsagittal plane as a straight line between the internal and external cervical os, even if the cervix appeared curved, using the echogenic line of the endocervical canal as a reference. Cervical volume was automatically calculated by the software using the cylinder formula (*V* = π × *r* ^2^ × *h*), derived from 2D ultrasound measurements, as demonstrated in Figure 1.

**FIGURE 2:**
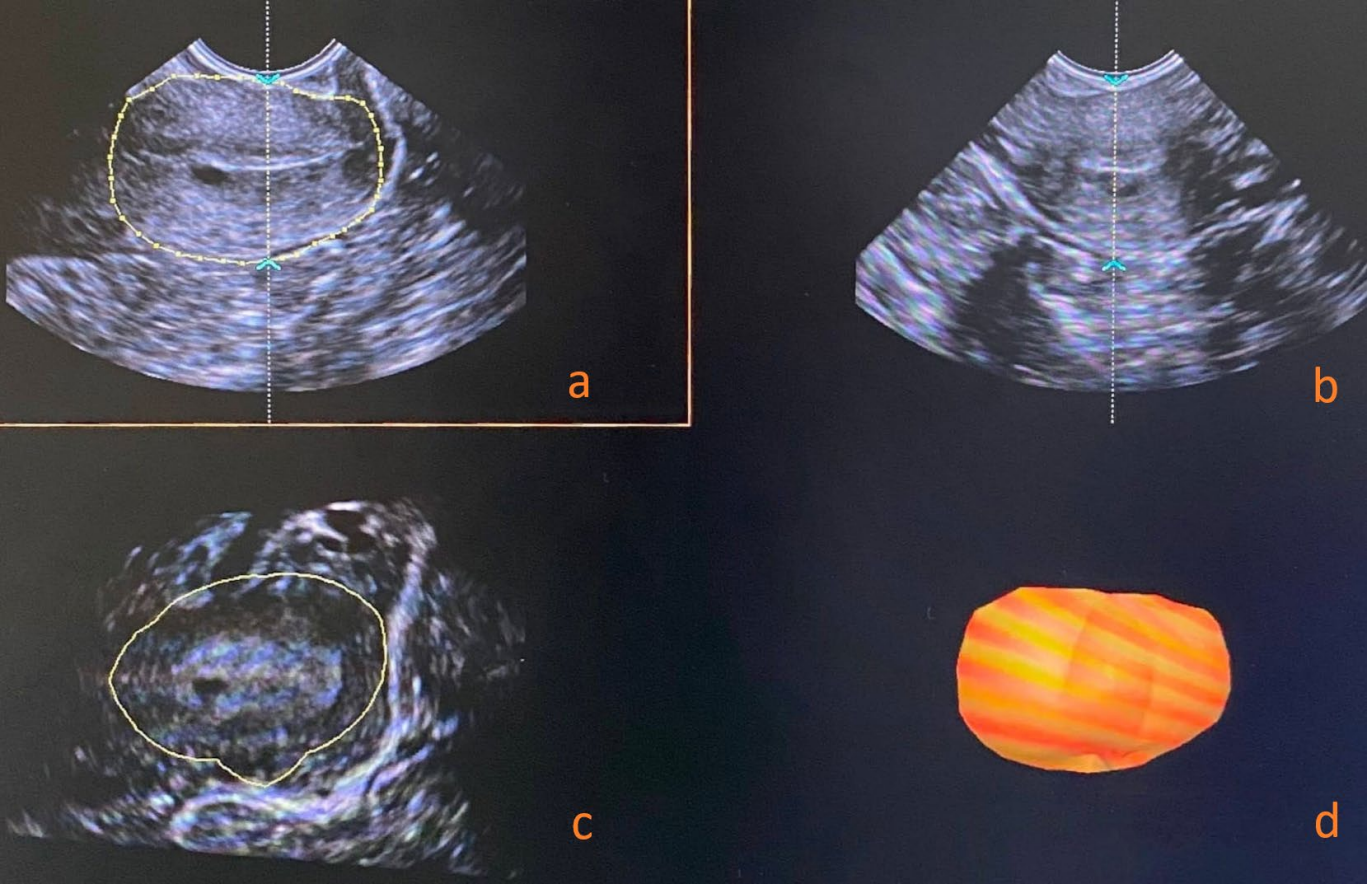
Cervical volume measurement using the VOCAL (Virtual Organ Computer‐Aided Analysis) software. (a) Reference 2D plane with cervical contour delineation for anatomical boundary definition. (b) Orthogonal 2D plane for spatial orientation and anatomical confirmation. (c) Rotational slices with sequential contouring at fixed angular increments for volumetric computation. (d) Final 3D reconstruction of the cervix generated from the serial contours.

The system was then switched to 3D mode. The cervical contour was manually traced in the A‐plane (sagittal), and after six steps, the VOCAL software (30° rotational step) generated the cervical volume (in cm^3^) (Figure 2).

All assessments were performed by the same two examiners, including one with over 30 years of experience in gynecologic ultrasound.

Patients who missed two of the three assessments were excluded from statistical analysis. Those who missed only one time point were retained and included in the corresponding pairwise comparisons. Sample size calculation was performed using the *pwr* package in R software, based on Cohen's *d* effect size derived from data reported by Nicolas et al.,^12^ assuming a statistical power of 90%. The required sample size was calculated to be 36 patients.

Changes in cervical length and 2D/3D volumes over time were analyzed in a single cohort. A one‐way repeated measures analysis of variance was performed using SPSS software (version 26.0, IBM Corporation, Armonk, NY, USA) to compare mean cervical measurements at three time points (baseline, 3 months, and 6 months), followed by Sidak's post hoc test for multiple comparisons. Effect size values (Cohen's *f*) were subsequently calculated using G*Power software (version 3.1, Heinrich‐Heine‐Universität Düsseldorf, Germany).

Proportional changes relative to baseline were assessed using Student *t* test, conducted in Matlab (version R2016a). Proportional values were calculated as (postoperative value/baseline value) × 100, and the proportion of the cervix remaining immediately after LLETZ was calculated by subtracting the excised specimen's length and volume from the corresponding baseline measurements. Generalized linear regression models were applied to assess the influence of the volume and length of the excised tissue, parity (grouped by nulliparous, one or two pregnancies, or three or more pregnancies) and loop electrode type (grouped by semilunar or triangular) on cervical regeneration. These groups were determined prospectively before data collection. No randomization was applied, and potential confounders such as previous cervical procedures were addressed through exclusion criteria. The intraclass correlation coefficient was estimated to assess concordance between 2D and 3D volume measurements using SAS software (version 9.4). A value of *P* less than 0.05 was considered statistically significant.

## 3 RESULTS

Eighty patients who were candidates for LLETZ were initially recruited. Twenty‐six of them did not meet the inclusion criteria: three patients were excluded because of previous excisional procedures, seven because of a diagnosis of cervical cancer, one because of pregnancy, three because the ultrasound images were not analyzable, 11 because they were lost to follow up, and one because of the use of a different type of loop, the rectangular loop. A total of 54 patients were included in the final analysis. Their ages ranged from 24 to 66 years, with a mean age of 39.1 years (standard deviation ±9.1 years). Half of the patients had one or two deliveries (27 patients; 50%), 20 (37%) were multiparous, and seven (13%) were nulliparous. Although HPV vaccination status was not assessed, it is likely that most participants had not been vaccinated, as the majority were outside the age range covered by the national immunization program at the time of vaccine implementation.

Histopathologic analysis of the excised specimens revealed that the majority corresponded to cervical intraepithelial neoplasia Grade III (CIN III) (34 patients; 63%), followed by CIN II (14; 25.9%), CIN I (three; 5.6%), invasive squamous carcinoma (two; 3.7%), and no atypia (one; 1.8%). In 39 cases (72.2%) the margins were negative, whereas 15 (27.8%) were positive. These results highlight the significant diversity of these measures. The length of the specimens ranged from 0.6 to 2.6 cm, with a mean of 1.64 ± 0.5 cm. The volume varied from 0.4 to 8 mL, with a mean of 4.24 ± 1.8 mL. These data are summarized in Table 1.

**TABLE 1:** Demographic and clinical characteristics of the study population.^a^

Variable	Value
Age, years	39 ± 9.10
Parity	
Nulliparous	7 (13.0)
Primiparous or secundiparous	27 (50.0)
Multiparous	20 (37.0)
Histopathology	
Negative	1 (1.8)
CIN I	3 (5.6)
CIN II	14 (25.9)
CIN III	34 (63.0)
Invasive	2 (3.7)
Loop used	
Semilunar	19 (35.2)
Triangular	35 (64.8)
Surgical margin status	
Positive	39 (72.2)
Negative	15 (27.8)
Excised specimen	
Length, cm	1.64 ± 0.5
Volume, mL	4.24 ± 1.8

Abbreviation: CIN, cervical intraepithelial neoplasia.

^a^ Data are presented as mean ± standard deviation or number (percentage).

### 3.1 Evolution of cervical dimensions

There was a significant reduction in cervical length between the initial measurement and the third month after LLETZ (from 32.30 ± 4.10 mm to 30.30 ± 5.90 mm, *P* < 0.001). However, when comparing the pre‐surgical measurement with that at 6 months, the cervical length returned to values close to baseline, with no significant difference (31.7 ± 5.10 mm, *P* > 0.05), suggesting complete cervical length regeneration.

**FIGURE 3:**
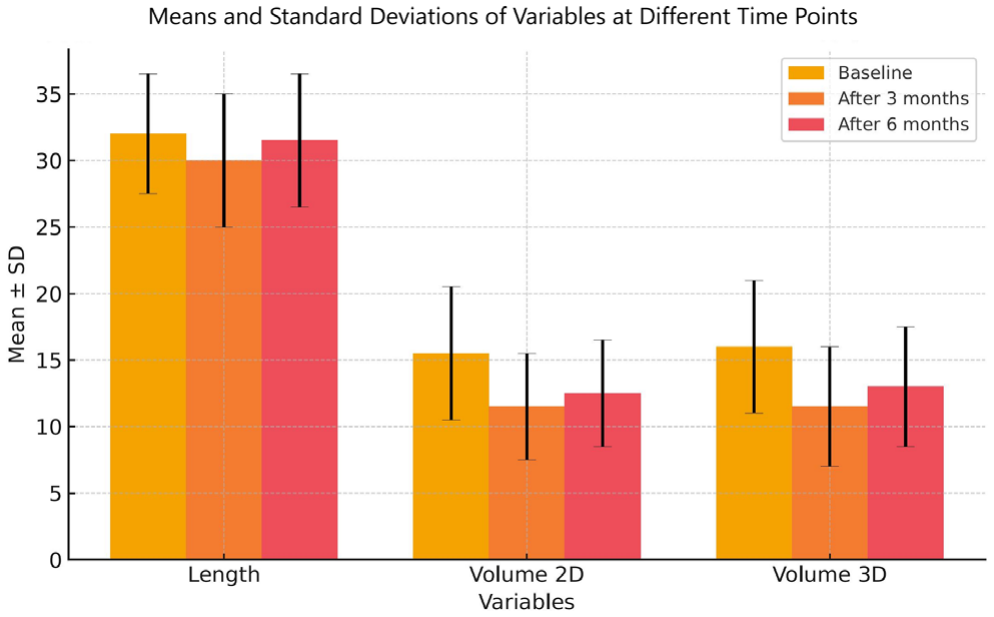
Evolution of cervical regeneration over time. Means ± standard deviations (SD) of cervical length and volume (two‐dimensional [2D] and 3D) at baseline, 3 months, and 6 months after large‐loop excision of the transformation zone. Error bars represent standard deviations.

In terms of volume, regeneration was less pronounced. The volume calculated using the cylinder formula increased from 11.58 ± 5.09 cm^3^ to 12.35 ± 4.17 cm^3^ between 3 and 6 months, but this difference was not statistically significant (*P* > 0.05). However, a significant reduction was observed when comparing the initial and 6‐month measurements (15.56 ± 5.41 cm^3^ to 12.35 ± 4.17 cm^3^, *P* < 0.001). Similarly, all VOCAL‐based measurements showed significant differences: 15.96 ± 5.30 cm^3^ at baseline to 11.77 ± 5.20 cm^3^ at 3 months and 13.11 ± 4.18 cm^3^ at 6 months (*P* < 0.001), indicating partial volumetric regeneration. The magnitudes of the differences between the measurements at the three time points were considered large (Table 2). Figure 3 graphically illustrates the evolution of these measurements.

**TABLE 2:** Comparison of cervical dimensions at baseline and consecutive postoperative evaluations.

Variable	Baseline	After 3 months	After 6 months	*P*‐value	ES
Length	32.30 ± 4.10^A^	30.30 ± 5.90^B^	31.7 ± 5.10^AB^	<0.001	0.41 (large)
Volume, 2D	15.56 ± 5.41^A^	11.58 ± 5.09^B^	12.35 ± 4.17^B^	<0.001	0.43 (large)
Volume, 3D	15.96 ± 5.30^A^	11.77 ± 5.20^B^	13.11 ± 4.18^C^	<0.001	0.43 (large)

*Note*: Data are presented as mean ± standard deviation. *P*‐values were calculated using one‐way repeated measures ANOVA with Sidak post‐test. Different superscript letters (A–C) indicate statistically significant differences between time points (*P* < 0.05). ES = effect size (Cohen’s f); DP = standard deviation.

Abbreviations: 2D, two dimensional; 3D, three‐dimensional; ES, effect size = Cohen's f.

**FIGURE 4:**
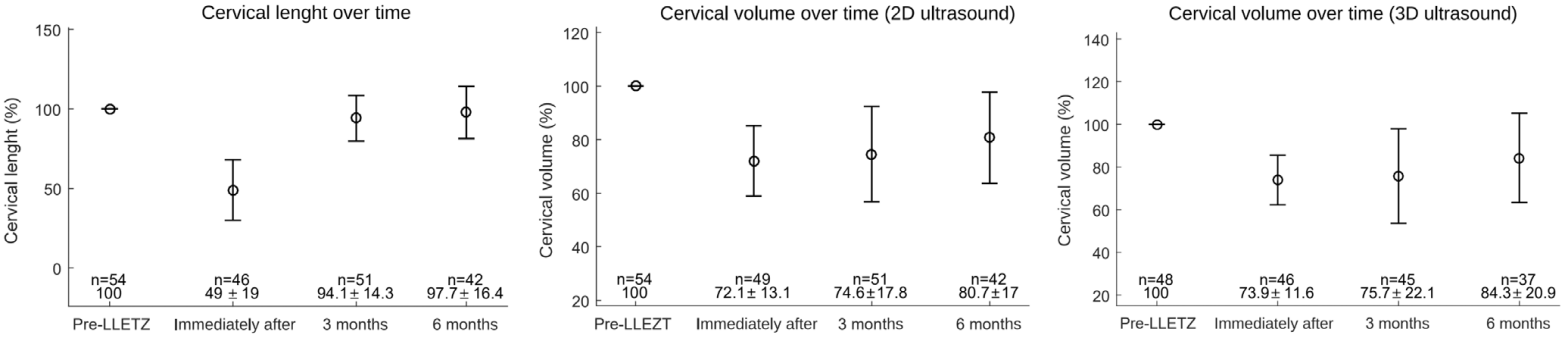
Evolution of the mean proportion of cervical length and volume relative to initial measurements. Values represent mean ± standard deviation of cervical length (% of initial length). Error bars represent standard deviations.

Cervical proportions relative to baseline values were also evaluated for both length and volumetric assessments, as shown in Figure 4. Cervical length demonstrated complete regeneration, with no statistically significant difference between the initial and final measurements (*P* = 0.369). In contrast, volumetric assessments indicated partial recovery, with significant differences for both 2D and 3D methods (*P* = 0.0001).

Data analysis revealed that most cervical length regeneration occurred within the first 3 months post‐excision, whereas cervical volume regeneration progressed more slowly and was more pronounced between 3 and 6 months (Figure 4).

### 3.2 Influence of variables on the cervical regenerative process

Parity, the type of loop used (semilunar or triangular), and the length and volume of the excised specimen were evaluated in relation to cervical regeneration 6 months after surgery.

For cervical length regeneration, the excised specimen volume showed a statistically significant correlation with recovery (*P* = 0.034).

Regarding cervical volume, parity was the only variable significantly associated with regeneration using the 2D method (*P* = 0.04). Higher parity was associated with lower regeneration rates, with nulliparous women showing the greatest regeneration (95.50%), followed by those with one or two deliveries (83.75%), Multiparous women had the lowest regeneration (72.57%). However, because of the marginal significance and limitations of the cylinder formula method, this finding should be interpreted with caution.

### 3.3 Correlation between ultrasound cervical volume measurements

**FIGURE 5:**
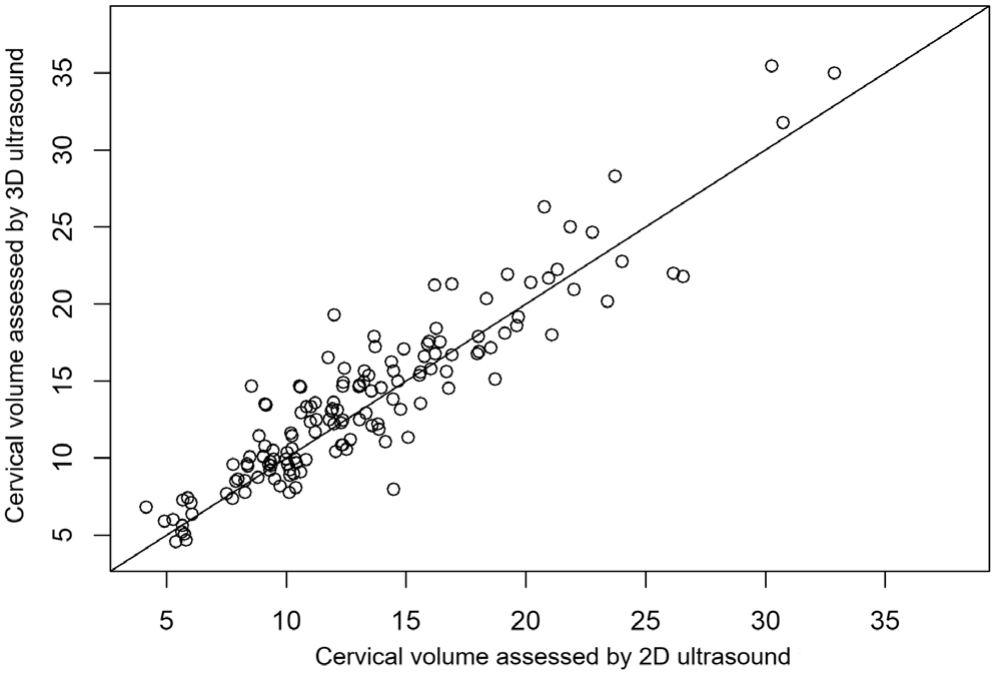
Correlation of cervical volumes measured using two‐dimensional ultrasound (cylinder formula) and three‐dimensional ultrasound (VOCAL [Virtual Organ Computer‐Aided Analysis] software).

The cervical volumes measured using the cylinder formula with 2D ultrasound and the VOCAL software with 3D ultrasound were correlated, as shown in Figure 5.

There was a high level of concordance (Intraclass Correlation Coefficient [ICC] = 0.92; 95% confidence interval 0.90–0.94).

## 4 DISCUSSION

Although LLETZ is widely used for the treatment of precancerous cervical lesions, its effects on cervical dimensions remain relatively underexplored. Methodological variations and individual anatomical differences present challenges to fully understanding the regenerative process. Some studies, including the present one, have compared postoperative cervical measurements to pre‐excisional dimensions.^13^, ^14^ Other research has focused on assessing changes within the crater resulting from the procedure.^14^, ^15^, ^16^ Additionally, Paraskevaidis et al.^17^ directly measured the dimensions of the crater, whereas others employed the tissue deficit, obtained from the difference between the initial and final cervical measurements, as a tool for indirect evaluation of the regenerative process.^13^, ^18^, ^19^

Our study findings are consistent with existing literature, indicating significant cervical regeneration within 6 months post‐LLETZ, with progressive increases in cervical dimensions. Cervical length fully recovered within 6 months, whereas volume reached over 80% of its original size, suggesting substantial, though incomplete, volumetric regeneration.

Gentry et al.^20^ reported complete cervical length regeneration 3 months post‐LLETZ, which aligns with our findings. Song et al.^13^ observed stable increases in cervical length and volume in 75 women, with length reaching 94.3% and volume 93.1% of baseline measurements at 12 months, with no significant differences after 6 months. Pinto et al.^14^ reported 96.8% length recovery and 98% volume recovery 6 months post‐LLETZ in 42 women.

Evaluating regeneration within the crater post‐LLETZ, Ciavattini et al.^16^ observed 89.5% length and 86.3% volume recovery. Pinto et al.^14^ reported similar values, with 91.1% and 91% recovery in length and volume, respectively.

In contrast, Nicolas et al.^12^ found a mean cervical length recovery of 71%, which remained significantly different from the initial measurement. Carcopino et al.^1^ also noted significant differences in cervical volume before and after LLETZ using 3D ultrasound.

The literature shows a large variability in baseline measures and regeneration rates. Papoutsis et al.^15^ reported volume regeneration ranging from 30% to 96% and length regeneration from 44% to 97%. In this context, identifying influencing factors is crucial. Most studies found no significant correlation between patient age and cervical regeneration post‐LLETZ.^12^, ^16^, ^21^ Other variables such as smoking status, histologic findings, lesion width and severity, surgical margin status, and parity also showed no correlation with regeneration.^12^, ^21^ In our study, parity influenced cervical volume recovery, with fewer pregnancies associated with greater regenerative capacity, probably due to better cervical tissue elasticity and integrity, and less cumulative tissue damage. However, this association should be interpreted with caution, given its marginal significance (*P* = 0.046) and the lack of significance when assessed using the VOCAL method.

Additionally, our study uniquely assessed the impact of different loop types (semilunar versus triangular) on cervical regeneration, and no significant differences were observed.

The literature presents conflicting results on the influence of excised specimen size on cervical regeneration. Nicolas et al.^12^ and Paraskevaidis et al.^17^ found no association between excised specimen length and tissue repair, suggesting that cervical tissue may compensate for loss. Conversely, our study found that specimen volume was associated with cervical length regeneration, which aligns with Ciavattini et al.,^16^ who reported that larger excisions reduce cervical length regeneration capacity. Founta et al.^19^ and Papoutsis et al.^15^ observed that higher proportions of excised volume correlate with lower regeneration rates, emphasizing the importance of limiting tissue removal for effective recovery.

Our research indicated that cervical length measurements were more reliable than volume measurements, consistent with findings reported by Pinto et al.^14^ Cervical volume measurements showed greater variability because of the complex calculations and higher susceptibility to ultrasonographic measurement inconsistencies. Calculating cervical volume using the cylindrical formula requires three measurements: length, anteroposterior, and transverse dimensions. Small deviations in each measurement can accumulate, leading to inaccuracies in the final volume calculations. Additionally, cervical morphology may not precisely match the cylindrical shape, introducing further bias and compromising the precision of results.

Although the 3D ultrasound volume measurement may offer improved accuracy, it also faces challenges. Similar to other researchers' experiences, this study found difficulty in delineating precise cervical boundary lines, potentially leading to the inclusion of non‐cervical tissue and the relative overestimation of cervical volume by VOCAL.^18^ Carcopino et al.^1^ reported low interobserver concordance before (ICC = 0.380) and after (ICC = 0.253) LLETZ in 3D ultrasound cervical volume assessment.

Comparing 2D and 3D ultrasound for cervical volume evaluation, our study corroborated the findings of other studies, demonstrating high concordance and correlation, which suggests that these methods are interchangeable.^14^, ^22^, ^23^, ^24^

Considering these facts, along with easier access, shorter examination times, and lower costs, 2D ultrasound cylindrical formula volume measurement is a viable and advantageous option for clinical practice. Nonetheless, considering the described difficulties in cervical volume measurement using both 2D and 3D methods, as well as its limited clinical routine application, the simplicity and precision of length measurement make it a more practical and reliable option for monitoring regeneration.

Currently, there is no evidence to support cervical dimension measurements in patients undergoing LLETZ. However, preoperative cervical length measurement may be valuable for women of childbearing age with future pregnancy plans. It can help surgeons choose the best approach and, combined with excised specimen evaluation, determine the proportion of cervix removed. This would enable additional prenatal care for women at high risk of adverse obstetric outcomes.

Another important aspect to be considered in the clinical management of these patients is HPV vaccination. Although it does not have a therapeutic effect on pre‐existing HPV infection or cervical neoplasia, HPV vaccination is associated with a lower rate of CIN recurrence and may contribute to a reduced risk of persistent or progressive disease after treatment. Therefore, it should be offered to eligible patients who have not yet completed the vaccination series, even in the post‐treatment setting.^25^ Several limitations should be acknowledged in this study. Quantifying cervical tissue loss during post‐excisional coagulation was not possible, and standardizing loop types was not feasible because of varying uterine cervical sizes and colposcopic findings. Sonographers were not blinded, and challenges in clearly delineating cervical boundary lines for VOCAL volume assessment and the internal cervical os for length measurement introduce subjective interpretation errors. Specimen measurements performed after formalin fixation may result in underestimation due to tissue contraction. Additionally, individual variability in regenerative capacity was not assessed.

In conclusion, progressive cervical regeneration was observed following LLETZ treatment. At 6 months post‐procedure, cervical length had fully recovered, and volume had regained over 80% of pre‐excisional measurements.

Parity influenced cervical volume regeneration, with fewer pregnancies being linked to greater regeneration. The volume of the excised specimen influenced the cervical length recovery. Given the increased risk of adverse obstetric outcomes with larger excisions or shorter cervices, a conservative approach is advisable for women of reproductive age.

Transvaginal ultrasound effectively assessed cervical dimensions, particularly cervical length. Both the 2D and 3D volumetric measurements showed good agreement, suggesting interchangeability.

## AUTHOR CONTRIBUTIONS

All authors contributed to the study conception and design. Material preparation, data collection and analysis were performed by FZS, HH, PM, MVBZ and SMQ. The first draft of the manuscript was written by FZS and all authors commented on previous versions of the manuscript. All authors read and approved the final manuscript.

## CONFLICT OF INTEREST STATEMENT

The authors have no conflicts of interest.

## Data Availability

Research data are not shared.
